# HIV and Tuberculosis Trends in the United States and Select Sub-Saharan Africa Countries

**DOI:** 10.3390/ijerph8062524

**Published:** 2011-06-23

**Authors:** Ousman Mahmud, Centdrika Dates, Luma Akil, Hafiz A. Ahmad

**Affiliations:** 1 Department of Biology, Jackson State University, 1400 J R Lynch Street, Jackson, MS 39217, USA; E-Mails: ousman.mahmud@students.jsums.edu (O.M.); drikaka05@yahoo.com (C.D.); 2 Biostatistical Support Unit, Department of Biology, Jackson State University, Jackson, MS 39217, USA; E-Mail: lumaakil@hotmail.com

**Keywords:** tuberculosis, HIV, United States, sub-Saharan Africa

## Abstract

Tuberculosis (TB) and Human Immunodeficiency Virus (HIV) are two catastrophic diseases affecting millions of people worldwide every year; and are considered to be pandemic by the World Health Organization. This study aims to compare the recent trends in TB and HIV in the United States and Sub-Saharan African Countries. Data (incidence, prevalence and death rates of HIV and TB) for the United States, Cameroon, Nigeria, and South Africa were collected from The Joint United Nations Programme for HIV/AIDS (UNAIDS), US Census Bureau and World Health Organization (WHO) databases and analyzed using Statistical Analysis Software (SAS v 9.1). Analysis of Variance (ANOVA) was performed to compare the variables of interest between the countries and across time. Results showed that percent rates of TB cases, TB deaths, HIV cases and HIV deaths were significantly different (*P* < 0.001) among these countries from 1993 to 2006. South Africa had the highest rates of HIV and TB; while US had the lowest rates of both diseases. Tuberculosis and HIV rates for Cameroon and Nigeria were significantly higher when compared to the United States, but were significantly lower when compared to South Africa (*P* < 0.001). There were significant differences (*P* < 0.001) in the prevalence of TB and HIV between the United States and the Sub-Saharan African countries, as well as differences within the Sub-Saharan African countries from 1993 to 2006. More analysis needs to be carried out in order to determine the prevalence and incidence of HIV and TB among multiple variables like gender, race, sexual orientation and age to get a comprehensive picture of the trends of HIV and TB.

## 1. Introduction

Tuberculosis and HIV are common and often deadly infectious diseases. Tuberculosis is caused in humans by mycobacteria, mainly *Mycobacterium tuberculosis*. The mode of transmission is through air that people, who has the disease, have coughed, sneezed, or spit. Half of the victims of this disease die because the infections are often asymptomatic and eventually progress to the active form without the proper treatment [1–4]. HIV is a lentivirus that causes acquired immunodeficiency syndrome (AIDS) in which the immune system begins to fail, leading to life-threatening opportunistic infections. Infection with HIV occurs by the transfer of blood, semen, vaginal fluid, pre-ejaculate, or breast milk. The modes of transmission are unprotected sex, contaminated needles, breast milk and transmission from an infected mother to her baby at birth [5–7].

Tuberculosis and HIV are considered pandemic by the World Health Organization (WHO). Although the incidence of TB cases in the United States is declining, the incidence in other parts of the World is increasing. Half of the reported cases in the U.S. occur in individuals that were born outside of the U.S. Studies have found that the HIV pandemic has increased TB prevalence globally, but its impact has been highest in developing countries in Africa, Asia, and Latin America [8,9]. Regions where doctors are confronted by drug-resistant tuberculosis provide sufficient evidence of the difficulty of treating both diseases [10]. Factors that contribute to the spread of tuberculosis in the U.S. and elsewhere include the increase in number of foreign born nationals, crowded living conditions, increase in drug resistant strains of tuberculosis, lack of access to medical care, and the increase in poverty [11].

South Africa is known to have the highest number of people infected with HIV, as shown in our data. It was reported that the former president of South Africa Thabo Mbeki had doubts about the causes of HIV. According to the reports, he did not believe that HIV was the exclusive cause of AIDS and argued for socioeconomic causes to be taken into considerations. Some of the social factors that play a role in South Africa being high risk are social inequalities in income, mobility-seasonal labor migrations and refugees, and sexual violence [12].

Tuberculosis is an important cause of mortality and morbidity in HIV infection in Africa and the leading cause of death among people living with HIV/AIDS [13,14]. Drug-resistant tuberculosis in Africa may be more prevalent than previously appreciated and is a serious problem for TB control programs in industrialized and developing countries alike [15]. In US, drug-resistant TB is relatively rare [16]. HIV/AIDS in the US is currently a disease of greater demographic diversity and has a comprehensive system of surveillance [17]. This study was conducted in order to compare the prevalence of Tuberculosis (TB) and Human Immunodeficiency Virus (HIV) in the United States and several Sub-Saharan countries from 1993–2006 in order to understand regional variations and coinfection trends of HIV and TB.

## 2. Methods

### 2.1. Data Collection

The HIV/AIDS data (total number at the end of each year of HIV cases and deaths) in the United States, Cameroon, Nigeria, and South Africa, were retrieved from The Joint United Nations Programme on HIV/AIDS (UNAIDS; http://www.unaids.org/en/default.asp). The HIV/AIDS data was part of the 2008 Report on the Global AIDS Epidemic. The Tuberculosis data (total number at the end of each year of new and relapse TB cases and TB deaths for each country) were obtained from the global tuberculosis database on the World Health Organization (WHO) website (http://apps.who.int/globalatlas/dataQuery/default.asp). The total population data (total population for each country at the end of each year) was retrieved from the US Census Bureau website (http://www.census.gov/). The tuberculosis and HIV data analyzed was over a period of thirteen years (1993–2006) and is current as of November 2009.

### 2.2. Statistical Analysis

The percent rate for each variable (HIV cases, TB cases, HIV deaths, and TB deaths for each country from 1993 to 2006) was calculated by expressing the ratio of the variable to the respective population. Data analysis was performed using the Statistical Analysis Software (SAS Inc, v9.1) and Microsoft Excel. Analysis of variance was performed using PROC GLM to determine the significant difference in the percent rate of cases and deaths for tuberculosis and HIV in the United States, Cameroon, South Africa, and Nigeria. The data was further classified using TUKEY standardized test. In addition, Microsoft Excel was used to generate bar graphs of the different variables for each country to represent the trends of HIV and TB for different countries over the thirteen years period.

## 3. Results and Discussion

### 3.1. Results

**Figure 1.**
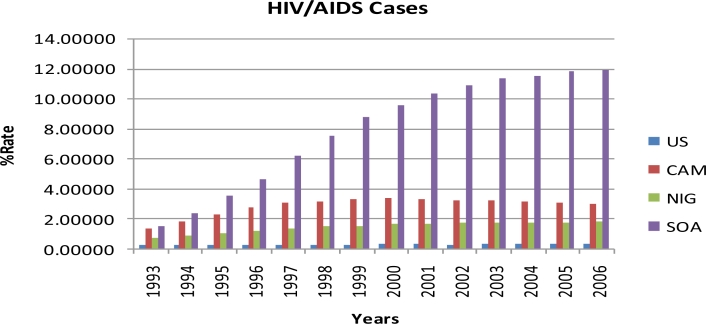
The percent rates of HIV/AIDS cases in the United States, Cameroon, Nigeria, and South Africa from 1993 to 2006 (US: United States, CAM: Cameroon, NIG: Nigeria and SOA: South Africa).

**Figure 2.**
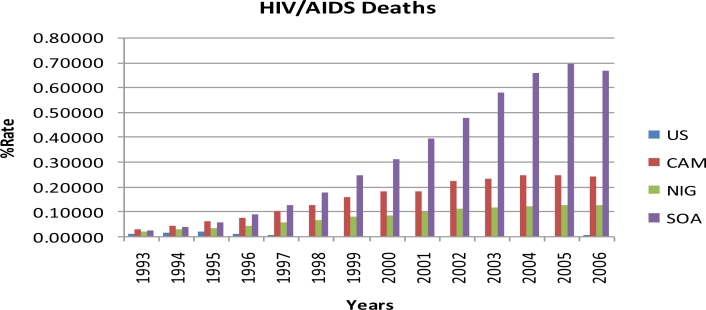
The percent rates of HIV deaths in the United States, Cameroon, Nigeria, and South Africa from 1993 to 2006 (US: United States, CAM: Cameroon, NIG: Nigeria and SOA: South Africa).

**Figure 3.**
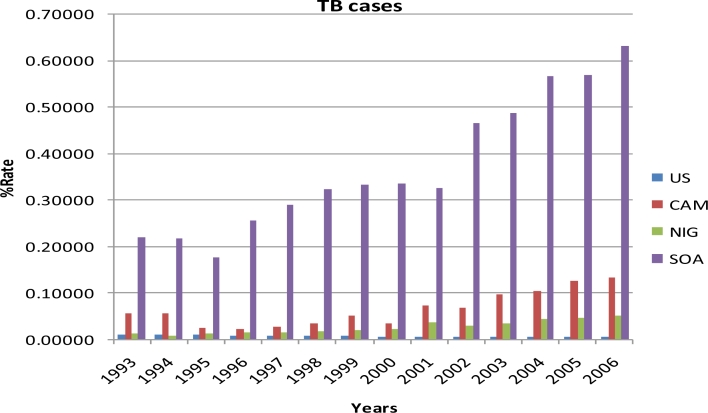
The percent rates of TB cases in the United States, Cameroon, Nigeria, and South Africa from 1993 to 2006 (US: United States, CAM: Cameroon, NIG: Nigeria and SOA: South Africa).

**Figure 4.**
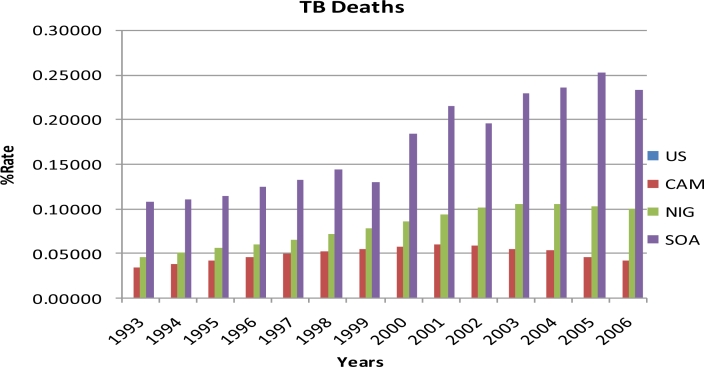
The percent rates of TB deaths in the United States, Cameroon, Nigeria, and South Africa from 1993 to 2006 (US: United States, CAM: Cameroon, NIG: Nigeria and SOA: South Africa).

Results of the study are summarized in Table 1 and Figures 1–4. Percent rates of HIV cases, TB cases, HIV deaths, and TB deaths were significantly different among the four countries (*p* < 0.0001). Sub-Saharan African countries had the highest percent rates of HIV and TB cases and deaths when compared to the United States. South Africa had the highest percent rates in all the four variables examined, i.e. HIV cases, TB cases, HIV deaths, and TB deaths, with over 100% from 1993 to 2006. United States had the lowest percent rates of HIV and TB cases and deaths when compared to the Sub Saharan countries from 1993 to 2006. Among the sub-Saharan African countries, Nigeria had the lowest percent rates of HIV and TB cases and deaths from 1993 to 2006 (Table 1).

TUKEY analysis of the percent rates of HIV cases revealed significant difference among all four countries, except for Nigeria, which was not significantly different from Cameroon and the United States (Table 1). South Africa had the highest percent rates of HIV cases among the four countries each year from 1993 to 2006. On the other hand, United States had the lowest percent rates of HIV cases among the four countries each year from 1993 to 2006. The percent rate of HIV cases in 2006 for South Africa (11.89342) was the highest percent rate from 1993 to 2006 among the four countries examined. The percent rates of HIV cases for Cameroon showed a decreasing trend after 2000, declining as much as 9% in 2006. The percent rates of HIV cases for United States also showed a decreasing trend after 2003, declining as much as 2% in 2006. Figure 1 shows the percent rates of HIV cases in the United States, Cameroon, Nigeria, and South Africa from 1993 to 2006.

Like HIV cases, the percent rates for HIV deaths were also significantly different among all the four countries, except for Nigeria, which was not significantly different from Cameroon and the United States using TUKEY analysis (Table 1). South Africa had the highest percent rates of HIV deaths among the four countries each year from 1996 to 2006. Cameroon had the highest percent rates of HIV deaths each year from 1993 to 1995 among the four countries. United States had the lowest percent rates of HIV deaths among the four countries each year from 1993 to 2006, declining as much as 68% from 1995 to 2006. The percent rate of HIV deaths in 2005 for South Africa (0.69498) was the highest percent rate from 1993 to 2006 among the four countries. The percent rates of HIV deaths for Cameroon, Nigeria, and South Africa showed decreasing trends from 2005 to 2006 whilst United States showed an increasing trend from 2004 to 2006. Figure 2 represents the percent rates of HIV deaths in the United States, Cameroon, Nigeria, and South Africa from 1993 to 2006.

The percent rates of TB cases were not significantly different among Cameroon, Nigeria and United States, but the percent rates for TB cases of South Africa was significantly different from the other three countries according to the TUKEY analysis (Table 1). South Africa had the highest percent rates of TB cases among the four countries each year from 1993 to 2006. United States had the lowest percent rates of TB cases among the four countries each year from 1993 to 2006, declining as much as 52%. The percent rate of TB cases in 2006 for South Africa (0.63247) was the highest percent rate from 1993 to 2006 among the four countries. The percent rates of TB cases for Cameroon, Nigeria, and South Africa showed an increasing trend from 2002 to 2006. Figure 3 presents a graphical view of the percent rates of TB cases in the United States, Cameroon, Nigeria, and South Africa from 1993 to 2006.

TUKEY analysis revealed the percent rates of TB deaths were significantly different among United States, Cameroon, Nigeria, and South Africa from 1993 to 2006 (Table 1). South Africa had the highest percent rates of TB deaths among the four countries each year from 1993 to 2006. United States had the lowest percent rates of TB deaths among the four countries each year from 1993 to 2006, declining as much as 65%. The percent rate of TB deaths in 2005 for South Africa (0.25184) was the highest percent rate from 1993 to 2006 among the four countries. The percent rates of TB deaths for Cameroon showed a decreasing trend from 2001 to 2006, declining by as much as 29%. Figure 4 represents the percent rates of TB deaths in the United States, Cameroon, Nigeria, and South Africa from 1993 to 2006.

### 3.2. Discussion

HIV is known to infect about 0.6% of the World population. From its discovery in 1981 to 2006, this infection leading to AIDS is known to have killed around 25 million people worldwide [18]. *Mycobacterium tuberculosis*, the main causative agent of TB, is thought to affect about a third of the World’s population [3]. TB is a particularly dangerous disease for persons infected with HIV and is the leading cause of death among people infected with HIV [19]. According to United Nations AIDS, since 1990, TB infection rates have increased 4-fold in countries that are heavily affected by HIV [20].

In this analysis, the prevalence of tuberculosis and HIV within the United States, Cameroon, Nigeria, and South Africa was elucidated. In South Africa, HIV deaths have a direct relationship to TB deaths based on percent rates from 1993 to 2006. South Africa had the highest percent rates of HIV cases and deaths among the countries examined, which could be attributed to these three factors: social inequalities in income and employment status, mobility, and sexual violence [12]. According to a report, despite being the most developed country and having the most advanced economy in Africa, South Africa has the highest number of HIV infection in the world [21].

The former president of South Africa Thabo Mbeki also contributed to South Africa’s high risk reputation for HIV. From the late 1990s to mid 2002, Thabo Mbeki had been open to the theories of some Western researchers who denied the causal relationship between the HIV virus and infection. He even invited some of them to a panel and had temporarily stopped all mother-to-child transmission prevention programs on the grounds that the medication used was harmful [22]. In most developing countries, knowledge resources are scarce, hence there is no conformation to universal precautions increasing the risks for the transmission of HIV and TB [23]. Unsafe practices such as using contaminated needles in health facilities also played a major role in the HIV status of South Africa. Health workers in public maternity and pediatric wards reused syringes under direct observation in 2005 and 30% of those surveyed did not see the need to use a new needle for each patient [24]. In Cameroon, the irregular use of gloves and other protective clothing for risky tasks, and recapping of needles after use were some of the risk factors identified for the HIV transmission in a hospital setting [23]. Poverty, stigma, discrimination, and a poorly coordinated health system constitute major risk factors for HIV treatment and prevention efforts in Nigeria [25].

The fact that West-Central Africa is an epicenter of the HIV pandemic and the emergence of anti-tuberculosis drug resistance, which is a serious problem for TB control programs, explains the high rates of HIV and TB cases and deaths in Nigeria and Cameroon when compared to United States (21496268) [15,26]. A recent study found that there is an emergence of drug-resistant *Mycobacterium tuberculosis* (MDR-TB) infections in Nigeria [27]. This has contributed to Nigeria showing an increasing trend in the percent rates TB deaths.

The decreasing trend in the percent rates of TB cases and deaths found in the United States could be attributed to the public health measures to control TB, which are currently focused on interrupting person-to-person transmission by promptly identifying and treating infectious patients and ensuring that they do not expose new contacts until treatment has rendered them noninfectious. The low percentages of rates of HIV cases and mortality in the United States could be attributed to the significant decline in HIV-associated mortality as a result of effective HIV treatment [28] and the success of the ABC (Abstinence, Being faithful, and Condom use) program.

Cameroon showed a decreasing trend in the percent rates of HIV cases and deaths; this can be attributed to its success in scaling-up access to antiretroviral therapy, effective HIV prevention programs, and effective prevention of mother-to-child transmission of HIV, by the National HIV/AIDS Control Committee that coordinate Cameroon national HIV/AIDS program, established in 1986 [29–32].

## 4. Conclusions

HIV and TB are major diseases that are pandemics in most parts of the World. Analysis of the data showed that the numbers of both diseases seems to have peaked and are declining in the four selected countries, indicating that there has been some success in combating these two deadly diseases. More analysis needs to be carried out in order to determine the prevalence and incidence of HIV and TB among multiple variables, such as gender, race, sexual orientation and age to get a comprehensive picture.

## Figures and Tables

**Table 1.** Mean and standard deviations of the percent rates of HIV and TB cases and death for USA, SA, Cameroon and Nigeria from 1993–2006.

**Variable/Country**	United States*	**South Africa**	**Cameroon**	**Nigeria**
HIV Cases	(0.3502 ± 0.0168)c	(7.9973 ± 3.6820)a	(2.9122 ± 0.6111)b	(1.5017 ± 0.3660)bc
HIV Deaths	(0.0097 ± 0.0056)c	(0.3258 ± 0.2509)a	(0.1559 ± 0.0800)b	(0.0833 ± 0.0387)bc
TB Cases	(0.0065 ± 0.0017)b	(0.3713 ± 0.1472)a	(0.0645 ± 0.0372)b	(0.0254 ± 0.0141)b
TB Deaths	(0.0004± 0.0001)d	(0.1722 ± 0.0538)a	(0.0499 ± 0.0080)c	(0.0806 ± 0.0217)b

* Variables with the same letter within a row are not significantly different.
